# Effects of oil and global environmental drivers on two keystone marine invertebrates

**DOI:** 10.1038/s41598-018-35623-w

**Published:** 2018-11-26

**Authors:** Maj Arnberg, Piero Calosi, John I. Spicer, Ingrid C. Taban, Shaw D. Bamber, Stig Westerlund, Sjur Vingen, Thierry Baussant, Renée K. Bechmann, Sam Dupont

**Affiliations:** 1 NORCE - Norwegian Research Centre – Environment, Mekjarvik 12, 4070 Randaberg, Norway; 2 Marine Biology and Ecology Research Centre School of Biological and Marine Science, University of Plymouth, Drake Circus, Plymouth, Devon PL4 8AA, UK; 3 Département de Biologie, Chimie et Géographie, Université du Québec à Rimouski 300 Allée des Ursulines, Rimouski, Québec G5L 3A1 Canada; 4 Department of Biological and Environmental Sciences (BioEnv), University of Gothenburg, The Sven Lovén Centre for Marine Infrastructure – Kristineberg, 45178 Fiskebäckskil, Sweden; 5 NOFO - The Norwegian Clean Seas Association for Operating Companies, P.O.box 8077, Forus 4068, Stavanger, Norway

## Abstract

Ocean warming (OW) and acidification (OA) are key features of global change and are predicted to have negative consequences for marine species and ecosystems. At a smaller scale increasing oil and gas activities at northern high latitudes could lead to greater risk of petroleum pollution, potentially exacerbating the effects of such global stressors. However, knowledge of combined effects is limited. This study employed a scenario-based, collapsed design to investigate the impact of one local acute stressor (North Sea crude oil) and two chronic global drivers (pH for OA and temperature for OW), alone or in combination on aspects of the biology of larval stages of two key invertebrates: the northern shrimp (*Pandalus borealis*) and the green sea urchin (*Strongylocentrotus droebachiensis*). Both local and global drivers had negative effects on survival, development and growth of the larval stages. These effects were species- and stage-dependent. No statistical interactions were observed between local and global drivers and the combined effects of the two drivers were approximately equal to the sum of their separate effects. This study highlights the importance of adjusting regulation associated with oil spill prevention to maximize the resilience of marine organisms to predicted future global conditions.

## Introduction

Humans are impacting life in the ocean in multiple ways^1^. Anthropogenic impacts can be both global and local. Global drivers are chronic, occurring over an extended period of time^2^. For example, as a consequence of increased atmospheric carbon dioxide (CO_2_), average surface temperature of the ocean is predicted to increase by 2–4 °C (ocean warming (OW)) and pH predicted to decrease by a further 0.3 to 0.5 units (ocean acidification (OA))^3,4^ by the year 2100^5^. Local drivers can also be chronic (e.g. nutrient input from rivers) but they may also be transient, brief and short lived (e.g. an acute oil spill)^2^. For example, in response to the worldwide demand for more petroleum products, the ongoing search by the oil and gas industry is extending geographically, particularly into the European Arctic from the north Atlantic into Greenland, northern Norway and northwest Russia^6,7^. Consequently, there is increased risk of acute oil spills from increased ship transport, operational discharges, and blow out scenarios as well as an increase in the geographical range and ecosystems over which potential accidental oil spills can occur.

Marine species and ecosystems will face multiple environmental challenges in the future ocean. While our knowledge of the biological consequences of local and global drivers separately is growing, their combined effects are still poorly understood^8–13^.

Responding to multiple environmental drivers may result in considerable alterations in the energy budget of an organism. An energy budget, or balance, is an account of how energy is obtained (i.e.through food consumption), against energy (1) not utilized (i.e. excreted as faeces), (2) used for respiration (routine maintenance metabolic costs), and/or (3) allocated to somatic growth/development and reproduction^14,15^. Some recent studies suggest that change in energy balance is a key factor in determining stress tolerance limits of an organism and that increased energy costs can directly translate into population- and ecosystem-level consequences^14^.

Both OA and OW have been shown to alter energy budget, metabolism and ultimately growth in many marine organisms including sea urchin and shrimp larvae^16,17^. Short-term oil exposure, on the other hand, tends to reduce larval activity which leads to reduced feeding (reduced assimilation of energy), impacting negatively on growth and ultimately mortality^18,19^. Therefore, to predict the effects of a local driver such as an oil spill, within the context of global changes (OA and OW), it is not sufficient to know the biological consequences of individual drivers. It is important to also investigate possible combined effects.

In the present study, we investigated the combined effect of global drivers (OA: ΔpH = −0.5 and OW: Δtemp = +3 °C) and a mechanically-dispersed crude oil exposure (0.5 mg L^−1^ nominal oil concentration) simulating the mechanical actions of waves on oil following a spill. The sensitivity and resilience to these drivers alone or in combination was investigated using larval stages of two keystone species: the northern shrimp (*Pandalus borealis*; stage I–IV larvae/zoea) and the green sea urchin (*Strongylocentrotus droebachiensis*; from zygotes to 6-arm pluteus, and from the 6-arm to 8-arm pluteus). Early life stages were chosen as they are considered to be particularly vulnerable to changes in environmental conditions^20^. A range of endpoints including growth, mortality, swimming, feeding and respiration were quantified. Our working hypothesis was that while exposure to global (OA/OW) and local (North Sea crude oil) drivers individually would significantly negatively affect larval early developmental stages, combined exposure would lead to a more severe impact than observed for single drivers. Furthermore, we hypothesized that the effects of both the global drivers (OA and OA and OW combined (OAW)) and local driver (Oil) can be explained by an alteration of the individual’s energy budget: OA/OAW by increasing costs to maintain homeostasis; oil through a narcotic effect.

## Results

### Seawater carbonate chemistry and temperature

Targets for the carbonate chemistry and temperature were achieved in all experiments (see Table S1 and Supplementary text for statistical analyses).

### Oil chemistry

In the shrimp experiment, the concentrations of total polycyclic aromatic hydrocarbons (PAHs) were 4.9 ± 0.1 and 5.5 ± 0.3 µg L^−1^ (means ± 1 sd) for Oil and OAW + Oil treatments, respectively (see Table S2). Approximately 90% of the PAHs in the exposure tanks were C0-C3 naphthalene’s, with the remaining 10% comprising of 3-ring PAHs and dibenzothiophenes (DBTs). For the urchin experiments, the concentrations of total PAHs were 4.8 ± 0.2 and 4.5 ± 1.2 µg L^−1^ for Oil and OA + Oil treatments, respectively. Total PAHs in control treatments were below the limit of detection (0.0025 µg L^−1^). Based on a total PAHs to oil ratio of 1%^19^, the oil concentration was estimated at 0.5 mg L^−1^.

### Mortality

**Figure 1:**
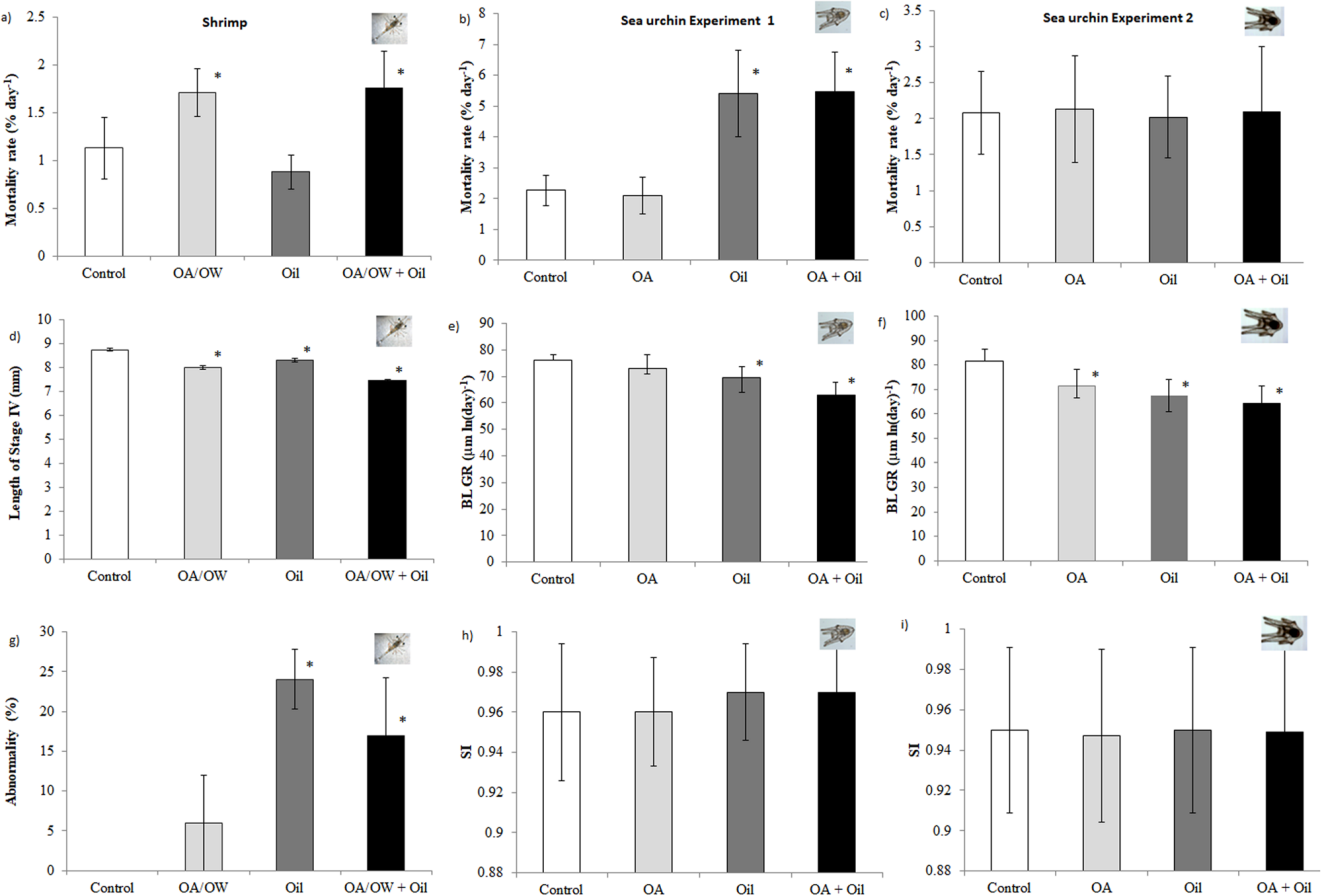
The effect of global drivers (pH and temperature) and Oil (crude oil conc. 0.5 mg L^−1^ on larvae of the northern shrimp *Pandalus borealis* (exposed to oil the day they hatched until 7 d post hatch and followed until all reached stage IV (day 27)) and the green sea urchin *Strongylocentrotus droebachiensis* (exposed to oil day 8–11(exp. 1 and followed from zygote to 6-arm pluteus) and 23–27 (exp. 2 followed until 8 arm pluteus)) days post fertilisation. (**a**–**c**) mortality rate; (**d**) length; (**e**,**f**) body length growth rate; (**g**) abnormality; (**h**,**i**) symmetry index. Control (pH 8.0, 6.7 °C, white), Oil (pH 8.0, 6.7 °C + Oil, dark grey), OA/OW (pH 7.6, 9.5 °C (for shrimp) and 6.7 °C (for sea urchins), ligth grey), OA/OW + Oil (pH 7.6, 9.5 °C (for shrimp) and 6.7 °C (for sea urchins) + Oil, black). Six replicates for each treatment. Values are presented as means ± SD.

Chronic exposure to global drivers (OAW, combined OA and OW) increased the mortality of shrimp larvae by 30% at the end of exposure, but not the acute exposure to Oil, and there was no significant interaction between Oil and OAW (Fig. 1a, see Table 1 and Table S3 for statistics). Exposure to OA did not affect mortality of sea urchin larvae. However, acute exposure to Oil resulted in high mortality for sea urchin larvae at early stage (Experiment 1, 8–12 days post fertilisation (dpf); Fig. 1b, Table 1 and Table S3), but not for later stage larvae (Experiment 2, 23–27 dpf; Fig. 1c, Table 1 and Table S3). There was no significant interaction between Oil and OA for larval urchin mortality (Table S3).

**Table 1:** The effect of global drivers (pH and temperature) and oil on larvae of the northern shrimp *Pandalus borealis* (exposed to oil the day they hatched until 7 d post hatch and followed until all reached stage IV (day 27)) and the green sea urchin *Strongylocentrotus droebachiensis* (exposed to oil day 8–11(exp. 1 and followed from zygote to 6-arm pluteus) and 23–27 (exp. 2 followed until 8 arm pluteus) days post fertilisation.

	Control	OA	%	P	Oil	%	P	OA + Oil	%	P
**Sea urchin experiment 1**										
Mortality rate (% day^−1^)	2.26 ± 0.49	2.11 ± 0.59	−7.14		**5**.**42** ± **1**.**40**	**138**.**76**	**0**.**003**	**5**.**47** ± **1**.**28**	**140**.**96**	**0**.**003**
BLGR (µm ln(day)^−1^)	76.13 ± 2.11	72.94 ± 5.43	−4.19		**69**.**37** ± **4**.**35**	**−8**.**89**	**0**.**014**	**63**.**05** ± **4**.**81**	**−17**.**18**	**<0**.**001**
SI	0.96 ± 0.03	0.96 ± 0.03	0.00		0.97 ± 0.02	1.04		0.97 ± 0.03	1.04	
**Sea urchin experiment 2**										
Mortality rate (% day^−1^)	2.08 ± 0.58	2.13 ± 0.74	2.50		2.02 ± 0.57	−2.88		2.10 ± 0.90	0.88	
BLGR (µm ln(day)^−1^)	81.54 ± 4.84	**71**.**33** ± **6**.**80**	**−12**.**52**	**0**.**010**	**67**.**62** ± **6**.**26**	**−17**.**07**	**0**.**002**	**64**.**34** ± **6**.**91**	−**21**.**09**	**<0**.**001**
SI	0.95 ± 0.04	0.95 ± 0.04	−0.32		0.95 ± 0.04	0.00		0.95 ± 0.04	−0.11	
Feeding (fluorescence exp^−1^)	0.08 ± 0.06	**0**.**05** ± **0**.**04**	−**43**.**26**	**0**.**001**	**0**.**02** ± **0**.**02**	−**77**.**66**	**<0**.**001**	**0**.**01** ± **0**.**01**	−**90**.**05**	**<0**.**001**
Activity (% active swimmers)	11.73 ± 11.11	**21**.**83** ± **13**.**02**	**86**.**18**	0.011	6.25 ± 4.54	−46.71		7.00 ± 3.80	−40.31	
Oksygen consumption (pmol O_2_ ind^−1^ h^−1^ mm^−1^BL)	2.56 ± 0.68	2.60 ± 0.45	1.56		1.82 ± 0.54	−28.91		2.94 ± 0.64	14.84	
	**Control**	**OAW**			**Oil**			**OAW + Oil**		
**Shrimp**										
Mortality rate (% day^−1^)	1.13 ± 0.32	**1**.**71** ± **0**.**25**	**51**.**33**	<**0**.**001**	0.88 ± 0.18	22.12		1.76 ± 0.38	55.75	<0.001
Length of stage IV (mm)	8.74 ± 0.07	**7**.**99 ± 0**.**07**	−**8**.**58**	**<0**.**001**	**8**.**30 ± 0**.**07**	−**5**.**03**	**<0**.**001**	**7**.**45 ± 0**.**06**	**−14**.**76**	**<0**.**001**
Abnormality (%)	0.00	6 ± 6	6.00		**24**.**04 ± 3**.**78**	**24**.**04**	**0**.**003**	**16**.**97 ± 7**.**30**	**16**.**97**	**0**.**025**
Feeding (artemia ind^−1^ h^−1^)	2.84 ± 0.30	3.56 ± 0.17	25.35		2.21 ± 0.41	−22.18		2.50 ± 0.23	−11.97	
Swimming (% where beam was broken)	0.90 ± 0.30	**6**.**63 ± 0**.**65**	**636**.**66**	**<0**.**001**	0.83 ± 0.75	−7.78		**7**.**46 ± 0**.**75**	**728**.**89**	**<0**.**001**
Oksygen consumption (nmole mgDW^−1^h^−1^)	62.76 ± 2.52	77.82 ± 4.96	23.99		51.9 ± 6.61	−17.30		71.37 ± 9.12	13.72	

For all treatments means ± standard deviation for each tested parameter are given. Significant treatments compared to the control are given in bold followed by a p-value from post-hoc test (Least Significant Difference (LSD)). % refers to percentage increase/decrease (−) in parameter compared to the control. Control (pH 8.0, 6.7 °C), Oil (pH 8.0, 6.7 °C + Oil), OA/OW (pH 7.6, 9.5 °C (for shrimp) and 6.7 °C (for sea urchins)), OA/OW + Oil (pH 7.6, 9.5 °C (for shrimp) and 6.7 °C (for sea urchins) + Oil). Six replicates for each treatment.

### Morphology

A significant (p = 0.002) proportion (20%) of the shrimp larvae exposed to Oil on its own and as OAW + Oil exhibited abnormal phenotypes (Fig. 1g, Table 1 and Table S3). However, there was no significant effect of global drivers (OAW) on abnormality of shrimps, and no interactions with oil. A 9% reduction in size in shrimp larvae was observed when exposed to OAW, and a 5% reduction when exposed to Oil. However, there was a 15% reduction in size when exposed to both stressors in combination (Fig. 1d, Table 1 and Table S3). Similarly, in sea urchin larvae, size reduction was greater when drivers were tested in combination (17 to 21%) than observed when they were tested individually (OA 11 to 14% reduction, Oil 6 to 9% reduction) (Fig. 1e,f, Table 1 and Table S3). No significant changes in symmetry index (SI) were observed among the urchin larvae (Fig. 1h,i. Table 1 and Table S3) for drivers singly or in combination.

### Feeding

**Figure 2:**
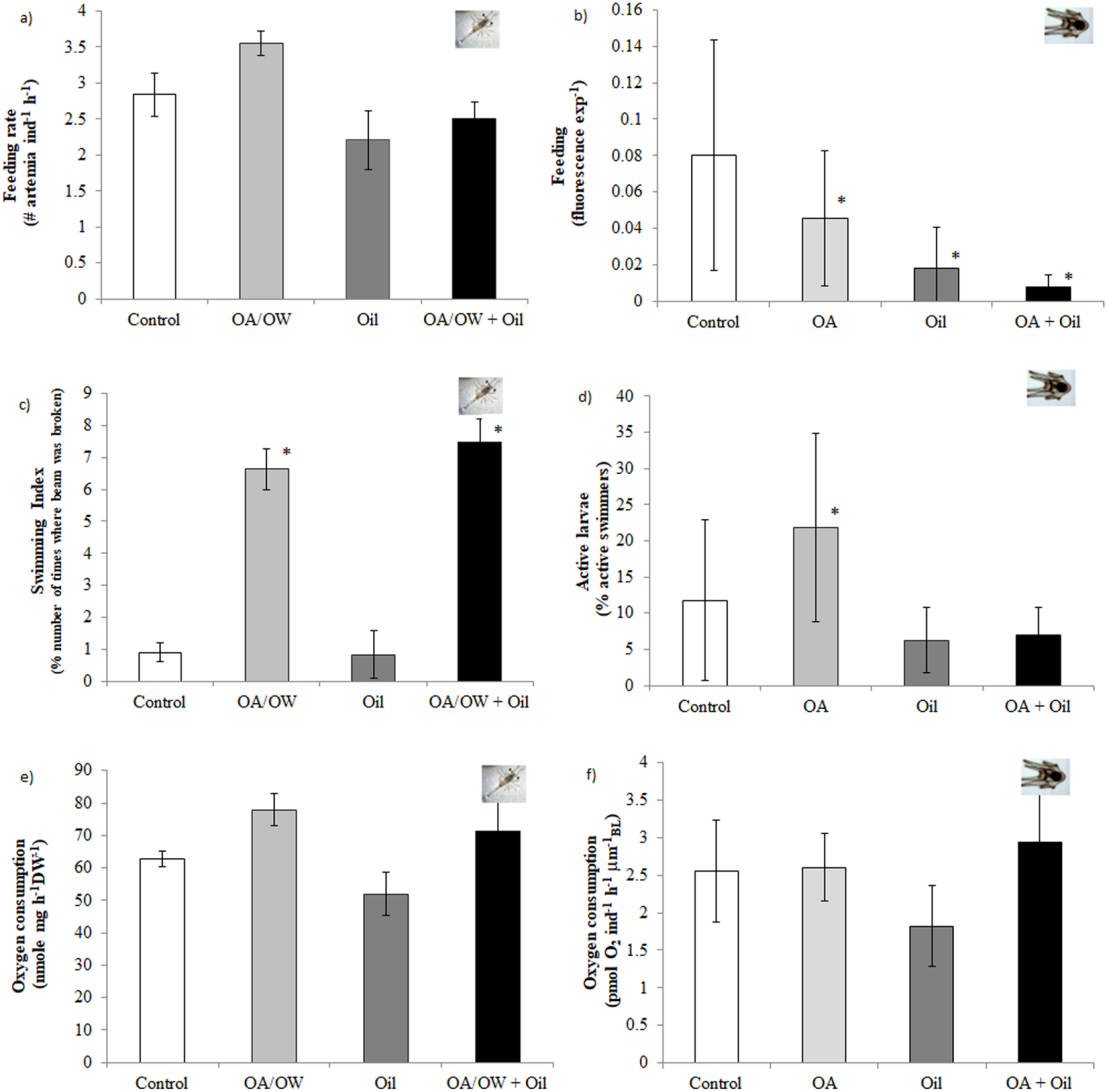
The effect of global drivers (pH and temperature) and Oil (crude oil conc. 0.5 mg L^−1^) on larval *Pandalus borealis* (exposed to oil the day they hatched until 7 d post hatch and followed until all reached stage IV (day 29)) and *Strongylocentrotus droebachiensis* (exposed to oil day 23–27, exp. 2 larvae were followed until 8 arm pluteus, 44 days post fertilisation. (**a**,**b**) feeding rate; (**c**,**d**) activity levels; (**e**,**f**) respiration rate. Control (pH 8.0, 6.7 °C, white), Oil (pH 8.0, 6.7 °C + Oil, dark grey), OA/OW (pH 7.6, 9.5 °C (for shrimp) and 6.7 °C (for sea urchins), ligth grey), OA/OW + Oil (pH 7.6, 9.5 °C (for shrimp) and 6.7 °C (for sea urchins) + Oil, black). Six replicates for each treatment. Values are present as means ± SD.

In shrimp larvae, the feeding rate (estimated as ingested Artemia h^−1^) was not influenced by the developmental stage (stage 3 and 4) (ANOVA 3, stage: F = 0.59, p = 0.45). Consequently, stages were pooled in subsequent analyses. Oil led to 1.2 times reduction in feeding rate but OAW or the combination of OAW and oil had no significant effect on feeding rates (Fig. 2a, Table 1 and Table S3).

In urchin larvae exposed early in development (8–12 days post fertilisation (dpf)), the feeding rate (estimated as fluorescence exposure times (exp)^−1^) was affected by Oil, but not by OA and there was no significant interaction between OA and Oil (Fig. S1, Table S3). During and after exposure to Oil, larval feeding rate was 5 times lower than the control (Fig. S1). For urchin larvae exposed later in their development (23–27 dpf), there was a significant effect of exposure to Oil and OA both singly and in combination (Fig. 2b, Table 1 and Table S3). Scheffe’s *post-hoc* test revealed that larvae raised in OA conditions ingested 1.8 times less food than the control. Moreover, exposure to oil alone (Oil) and in combination with OA (OA + Oil) resulted in a 6.2 times reduction in feeding rate compared with the control (Fig. 2b).

### Metabolic rate and activity

There was a significant Oil-related increase in the proportion of non-swimming shrimp larvae by 54% compared to the control (Fig. S2, p = 0.001). The swimming index calculated for stage 3 larvae increased significantly by 8.1 times in larvae exposed to OAW but was not impacted by oil or their interaction (Fig. 2c, Table 1 and Table S3). No significant effect of oil, OAW singly or in combination on metabolic rate was detected (Fig. 2e, Table 1 and Table S3). Exposure to OA made no significant difference to the swimming of sea urchin larvae (23 dpf) so data were pooled from experiments on effect of Oil, singly or in combination with OA. 34% of the larvae were not swimming in oil treatments as compared to 4% in larvae in the control (Fig. S3). However, for a later developmental stage (31 dpf), both OA and Oil had significant effects on swimming but not in combination (Fig. 2d, Table 1 and Table S3). The percentage of active larvae was greater in OA compared to the control (Fig. 2d). No significant effects of OA, Oil or their interaction were detected for metabolic rate, which increased linearly with body length (BL) (Fig. 1f, Table 1 and Table S4).

## Discussion

Both shrimp and urchin larvae were negatively impacted by OA and OAW drivers resulting in decreased growth, suggesting a trade-off between energy requirements for maintenance activities and for growth as has been shown before^14,15,21–24^. For example, Stumpp *et al*.^25^ found that exposure to OA reduced larval growth in a sea urchin larva because of a trade off with increased maintenance costs. Stumpp *et al*.^16^ also showed a reduction in gastric pH in larvae exposed to OA. This resulted in reduced digestive efficiency, issuing in compensatory feeding. They argued that despite such compensation, the larval energy budget was still significantly impacted by increased costs for gastric alkalization and food capture, resulting in reduction in growth. This is supported by our study as the chlorophyll a-dependent fluorescence intensity was significantly reduced by 28–40% in sea urchin larvae (Experiment 2) suggesting a decreased digestion efficiency.

Acute exposure to oil also reduced growth, feeding and activity in larvae of both species as well as resulted in an increase in abnormal phenotypes of shrimps and increased mortality in sea urchins. Similar oil-related reductions in swimming and feeding have been observed for adult and naupliar copepods, and krill larvae^19,26^ and as well reduced growth rates in larval shrimp^27^ and herring^28^. The reduction in swimming activity and feeding efficiency during oil exposure observed here may be due to a non-specific narcotic effect of the most polar compounds of the oil (such as low log*Kow* PAHs and BTEX benzene, toluene, ethylbenzene and xylenes) inducing incoordination and unresponsiveness to external stimuli or alteration of digestive cell membrane function^26^. Hydrocarbon mixtures found in crude oil can also induce non-polar narcosis, which results in the alteration of cell membrane function^29^. PAHs and BTEX, can readily be partitioned into the cellular membranes of invertebrates depending upon their log *Kow*^30^. This can result in mild toxic effects or even mortality, depending upon the level of exposure^30,31^. The reduced feeding efficiency observed under oil exposure could explain the lower growth rates observed in both species.

In both species, feeding rate returned almost to control levels post oil exposure. This has also been observed for copepods^32^. One effect of oil exposure is to reduce food (and hence energy) intake. This is important as any fitness costs incurred will be highly dependent on an organism’s energy reserves. Later developmental stages may possess greater energy reserves and, as a result, be more able to cope with transient food shortages^33^. This could explain our observation that later developmental stages of the urchin were not as negatively impacted by oil exposure as earlier stages. This highlights the importance of considering several developmental stages and species when investigating the impacts of stressors to identify factors limiting the generation turnover and life-history bottlenecks^20,24^.

Responding to multiple stressors can result in considerable energy expenditure by an organism as it can involve multiple molecular and cellular signaling pathways (i.e. modes of action)^9^. We hypothesized that the effects of both the global drivers (OA and OAW) and local driver (Oil) can be explained by an alteration of the individual’s energy budget: OA/OAW by increasing costs to maintain homeostasis; oil through a narcotic effect. Consequently, we also hypothesized that when combined, these two categories of stressors may result in an even greater negative impact on fitness (increased abnormality, decreased growth). This is supported by this present study. Interestingly, no statistically significant interaction was observed between the chronic global and acute local drivers. The combined effects were approximately equal to the sum of their separate effects for the scenarios tested. For example, the impact of the combined drivers on growth rate could be predicted by the sum of the observed impacts on each driver alone. Unfortunately, our experiments as they only encompassed two scenarios for each tested category of drivers do not allow us to resolve how these stressors interact. That would require resolving performance curves for each individual driver together with a better understanding of their mode of action^8^.

Both global and local drivers had negative consequences on growth rates. This can translate into negative consequences for larval survival in the field as larvae would spend longer time in the plankton before settlement, increasing their vulnerability to predation^34^. This could be further enhanced because of the reduced larval activity resulting from the narcotizing effects of oil and may also have consequences for successful dispersal and recruitment^35,36^.

The present study warrants considering local drivers such as oil exposure from spill situation as well as other contaminants for the adaptation to and management of global drivers (e.g. OA and OW). In fact, an effective management of local drivers, such as oil contamination ultimately impacting similar physiological process (such as energy budget) than those encompassing global drivers, could mitigate the detrimental impact of future global environmental changes^37^. This in turn would empower and encourage local decision makers to act on local driver regulations, and by doing so increase the environment resilience of natural populations from the negative impacts of future global drivers^38^.

## Methods

### Specimen collection, spawning and larval maintenance

Adult green sea urchins were collected by hand by SCUBA divers in Lysefjorden, Norway, from 15 m depth in mid-February 2011. Spawning was induced in April 2011 by intracoelomic injection of 0.5 mmol.L^−1^ potassium chloride (KCl) in filtered sea water (FSW). In each experiment, eight females and three males were used. Zygotes were allowed to divide once before being pooled and then transferred to numerous separate aquaria. After 5 d, larvae were fed daily (concentration 150 µg C L^−1^, with an initial algal density approximately 6,000–7,000 cells mL^−1^ and mean size 7.5 µm ± 0.8 µm) with the cryophyte algae *Rhodomonas* sp until the experiment finished. During this time, they were also fed with the algae *Isocrysis*.*sp* (concentration 150 µg C L^−1^, with an initial algal density approximately 186,000 cells mL^−1^ and mean size 4.5 µm ± 0.5 µm).

Forty-eight ovigerous northern shrimp were acclimated for two weeks to laboratory control conditions (T = 6.7 °C, S = 33, pH = 8.05). Ovigerous females (N = 24 in total, six *per* treatment) were transferred to individual aquaria (vol. = 9 L) supplied with flow-through sea water. Aquaria were checked daily for hatchlings. When noticed, hatchlings were removed from these holding tanks and batches of larvae (N = 200) from the same female were kept in the same individual glass aquaria exactly as described in Arnberg *et al*.^19^. From each experimental treatment six replicates (batches from six different mothers) were exposed and monitored until they reached stage IV zoea. Post-hatch shrimp larvae were fed *ad libitium* with freshly hatched *Artemia salina* nauplii (*Artemia* length 450–550 µm, feeding density 1,000 indiv L^−1^) twice a day (morning and afternoon) for the entire duration of the experiment. For the first week, larvae were also fed with phytoplankton *Thalassiosira weisslogi* 1200^TM^ (Microalgae, Vigra, Norway, 2 × 10^4^ cells L^−1^) once a day.

### Experimental setup

Two experiments were performed on larval stages of the green sea urchin in April 2011 (duration Experiment 1: 18 d, Experiment 2: 44 d). Sea urchin embryos and larvae were raised into two different pH treatments: a control = pH_NBS_ 8.0 corresponding to the average pH experienced by larvae at present, and OA = pH_NBS_ 7.6, the average pH that is projected for 2100^36^. For each pH, six flow through header tanks were supplying 12 replicates conic aquaria (vol. = 13 L, flow: 100 mL min^−1^). A gentle bubbling with air stones was created to keep the larvae in suspension. There was a total of four treatments with six replicate aquaria for each treatment, Control (pH_NBS_ 8.0, no oil), OA (pH_NBS_ 7.6, no oil), Oil (pH_NBS_ 8.0, oil) and OA + Oil (pH_NBS_ 7.6, oil) (See Fig. S4 for more information on the experimental design).

For the shrimp experiment, larvae were raised in two different combinations of pH and temperature levels using a collapsed design: a control, pH_NBS_ 8.0 and 6.7 °C, corresponding to the pH and temperature experienced by larvae at present, and OAW, pH_NBS_ 7.6 and 9.5 °C, as the average pH and temperature projected for 2100^1–4^. Shrimp larvae were kept in two separate continuous flow systems, consisting of six header tanks (vol. = 12 L, flow = 1 L min^−1^) where water temperature was regulated at 6.7 ± 0.08 and 9.5 ± 0.07 °C respectively, using heat exchangers. Header tanks delivered sea water to 24 × 18 L aquaria each containing 200 shrimp larvae. There was a total of four treatments with six replicate aquaria for each treatment, Control (pH_NBS_ 8.0, 6.7 °C, no oil), OAW (pH_NBS_ 7.6, 9.5 °C, no oil), Oil (pH_NBS_ 8.0, 6.7 °C, oil) and OAW + Oil (pH_NBS_ 7.6, 9.5 °C, oil) (See Fig. S5 for more information on the experimental design).

#### Larval exposure to oil

Sea urchin larvae were continuously exposed for 4 days to a nominal concentration of 0.5 mg L^−1^ crude oil using a flow-through system creating a mechanical dispersion mimicking that created under the actions of waves on oil after a spill. No dispersants were used. Two different experiments were performed: (i) In the first experiment, sea urchin larva was exposed to oil between 8–11 dpf and larval response was followed in clean seawater until day 18 dpf. In this experiment larvae were followed from zygotes to 6-arm pluteus larvae and the larvae were exposed to oil during the 6-arm pluteus stage. (ii) At the end of this experiment, each culture that was not exposed to oil (n = 6) was split into two replicated cultures for a total of 12 replicates per tested pH. Half of the replicates were exposed to oil at 23–27 dpf and larval responses were further monitored in clean seawater until day 44 dpf (experiment 2). In this experiment, larvae were followed from the 6-arm pluteus stage to 8-arm pluteus stage just before settlement, and larvae were exposed to oil during the 6 arm pluteus stage.

Likewise, shrimp larvae were exposed continuously to a nominal concentration of 0.5 mg L^−1^ crude oil as above, during 7-days. Shrimp larvae were exposed to oil from the day they hatched until 7 d post hatch (dph). Larval responses were further monitored in clean seawater until day 27 when most of the shrimp larvae developed into stage IV (day 27 at control conditions and day 19 at OAW conditions).

### pH manipulations, oil manipulations, oil and carbonate chemistry and physical measurements

In both experiments a crude oil from the North Sea Troll field was used (NOFO, Norwegian Clean Seas Association for Operating Companies). An oil-water dispersion (OWD) was generated continuously using addition of oil in a flow-through system with a mixing valve resulting in a mechanical dispersion of oil with a mean droplet size of 12 μm in the seawater. Such system results in the exposure with the organisms to a combination of dispersed and dissolved components of oil depending on their water solubility (log *Kow*). See Supplementary Fig. S6 and text for more information.

### Mortality

Larval cultures were monitored daily. For sea urchins, one subsample (exp. 1) or two subsamples (exp. 2) of 10 mL were collected from each culture, counted and fixed in buffered 4% paraformaldehyde (PFA) in FSW for subsequent analysis. Mortality (% day^−1^) was estimated as the regression coefficient of the linear regression between relative density (%) and time. This relationship was significant for 21 of the 24 cultures (p < 0.05, see Tables S5 and S6). For shrimps, dead larvae were counted days 6, 9, 13, 17, 19 and 27 allowing the calculation of the relative mortality (%) from initial larval density. The sampling time was based on data from a previous study on the combined effects of elevated temperature and low pH on the developmental physiology of Northern shrimp^17^. Since there was a reduction in development time for the larva raised in the OAW conditions, they were sampled on the same day to determine differences in mortality between the treatments. Mortality rate was also calculated as the slope of the linear relationship between density and time (p < 0.01; see Table S7). The mean mortality rate (MR in % d^−1^) was calculated from individual mortality rates within each treatment.

### Morphometrics

For sea urchins, 10–15 larvae in each replicate were photographed every other day with a digital camera mounted on a dissecting microscope (x5–20 mag, depending upon stage) using polarized light to visualize the skeleton. Three morphometric parameters (body length (BL) and posterolateral rod lengths (POL1, POL2)) were measured for each larva using Image J software. Body length growth rates (BL GR in μm ln (d)^−1^) were calculated from the slopes of the significant logarithmic relationship between BL (μm) and time (d) (see Tables S8 and S9). Symmetry index (SI) was calculated for each larva as

1 $${\rm{SI}}=\frac{Min(POL1,POL2)}{Max(POL1,POL2)}.$$

For shrimps, total length (TL) was measured for a selection of 6–13 individuals *per* replicate. Morphological classifications (i.e. presence of abnormalities) of abdominal sixth segment of the stage IV shrimp larvae were also performed; the larvae sixth elements were classified as normal or abnormal (missing endopodite or exopodite or unsymmetrical). An abnormality index was calculated as the percentage of abnormal larvae.

### Feeding

Fluorescence microscopy was used to assess the algal content in the digestive gland of sea urchin larvae. One hour after feeding, larvae were collected from each replicate and larvae pictures obtained using a Zeiss Axioplan 2 imaging epifluorescence microscope (bifocal (5X)) fitted with an Axiocam MRc5 camera, with a red fluorescent rhodamine filter. Semi-quantitative measurements of algal presence in the digestive system was carried out by quantifying the saturation of red fluorescence using the Axiovision software. For each larva, body length (µm), area of larval stomach and stomach fluorescence were automatically measured (Fig. S7a,b). Stomach fluorescence was calculated as red fluorescence *per* unit time (fluorescence ms^−1^). Feeding was assessed between 12–15 dpf in experiment 1 and between 23–33 dpf in experiment 2. For the shrimp experiment, we used a modification of the clearing rate methods described in^10^. Five larvae at the same developmental stage were transferred into 1 L Schott bottles, placed in a temperature-controlled environment (temp = 6.7 or 9.5 °C) and starved for 24 h before start of the test. Freshly hatched *Artemia* nauplii (initial prey conc. 150 indiv L^−1^) were then added and the bottles were incubated for 6 h. After the incubation period, shrimp larvae were carefully removed and the water containing *Artemia* nauplii was sieved using a 40 µm BD Falcon^TM^ cell strainer (BD Biosciences, Franklin Lakes, USA). The remaining nauplii were counted and feeding rate was expressed as number of prey consumed *per* individual shrimp larvae *per* unit time (number of *Artemia* indiv^−1^ h^−1^).

### Metabolic rate and activity

Sea urchin larvae activity was assessed visually at day 23 during the oil exposure. For each culture, a sample of at least 20 larvae was placed in a Petri dish (diameter: 4 cm) and larvae were scored as active or passive (not swimming actively, only at the bottom of the Petri dish with no or little movement). Furthermore, a swimming activity test modified from^11^ was performed at 31 dpf. For each replicate, 15 larvae were transferred to 2 mL glass vials with treatment sea water (pH 8.1 or 7.6). Presence or absence of food (300 µg C L^−1^ *Rhodomonas* sp.) were compared. The syringe chamber was filled with 2 mL of sea water with and without algae and was connected to the glass vial with a silicon tube. After 24 h larvae that had entered the syringe chamber were counted as “active swimmers”.

Rates of oxygen uptake (as a proxy for metabolic rates) for sea urchin were measured, using a custom-built closed, glass-bottle respirometer (vol. = 5 mL) fitted with airtight rubber fitting into which O_2_ electrodes had been inserted. 200 sea urchin larvae were placed in the incubation chambers filled with sea water at the appropriate pH and sealed with an airtight stopper before submersion in water baths to maintain constant temperature. Measurements of dissolved O_2_ concentration were performed every 2 sec for the entire duration of the incubation (approx. 48 h) using O_2_ electrodes (1302, Strathkelvin Instruments, Glasgow, UK coupled to a multichannel O2 meter (928, Strathkelvin Instruments, Glasgow, UK). Continuous measurements were conducted to demonstrate linearity of pO_2_ decline in the respiration chambers during the incubation. At the end of each trial, viable larvae were counted (mean 0.8 ± 2.5% were dead). Rates of oxygen uptake were corrected for the number of living larvae left in each respirometer and for background respiration (incubation with no larvae present).

Two swimming tests were carried out for the shrimp larvae. The first was made during oil exposure according to Larsen *et al*.^39^. For the second one, ten to fifteen larvae were placed into 1 L glass beakers. An Infrared light emitting diode was aligned with a phototransistor on opposite sides of the glass beaker. The width of the infrared light beam passing through the water, together with the surface area of the phototransistor open to this light, was controlled using baffles pushed into the support pillars in front of the phototransistor and emitter. As larvae swam through the light beam, breaking its path, the reduced amount of light, falling onto the phototransistor caused a drop in the output voltage. The white light emitting diode was positioned directly above the phototransistor, as light source towards which the larvae swam. Observations had shown that larvae swam upwards into the light then ceased swimming, falling slowly back down before recommencing swimming back into the light. This behavior allows response to light to be measured as the intensity of repeated swimming activity recorded as the number beam breaks per hour. Voltage output from the phototransistor was logged at intervals of 0.2 sec throughout the one-hour test periods using a data logger (NI USB - 6009, National Instruments, Texas, USA). Metabolic rates for shrimp were measured, using the method by Arnberg *et al*.^17^.

### Statistical analyses

Statistical analyses were performed using the SAS software. All endpoints (MR in % day^−1^, BL GR in μm ln (day)^−1^, SI, abnormality index and, shrimp larval length in mm) were analysed with a two-way analysis of variance (ANOVA) to test for significant differences between acute driver (oil), chronic drivers (OA or OAW) and their interaction. Data were normally distributed according to the Kolmogorov-Smirnov test, and when not, were log transformed. Equality of variance was tested using the Levene median test. The significance level α was set at 0.05.

## Electronic supplementary material

Supplementary Information

## Data Availability

The datasets generated during and/or analysed during the current study are available from the corresponding author on reasonable request.
