# Supplementary material for: Effects of oil and global environmental drivers on two keystone marine invertebrates
Source: Sci Rep. 2018 Nov 26;8:17380. doi: 10.1038/s41598-018-35623-w (PMC6255813; doi:10.1038/s41598-018-35623-w)
Supplement: Supplementary file 1 — Supplementary Information [file 41598_2018_35623_MOESM1_ESM.docx]

Effects of oil and global environmental drivers on
two keystone marine invertebrates

Maj Arnberg, Piero Calosi, John I. Spicer, Ingrid C. Taban, Shaw Bamber, Stig Westerlund, Sjur Vingen, Thierry Baussant, Renée K. Bechmann, Sam Dupont

**Methods**

**pH manipulations, oil manipulations, oil and carbonate chemistry and physical measurements.**

pH and oil manipulations

pH was directly manipulated in each replicate using pH-controllers (AB Aqua Medic GmbH pH computer, Bissendorf, Germany) connected to a solenoid valve regulating addition of pure CO_2_ gas. Seawater pH was measured every 5 min on the NBS scale using a pH probe (Orion Star Plus™ 3-Star and Ross^®^ Electrodes, Thermo Fisher Scientific Inc, Beverly, USA) coupled to a calibrated pH meter (Orion Star Plus™, Thermo Fisher Scientific Inc) and a multi-channel datalogger (D-130, Consort, Turmhout, Belgium) using the Star Plus Navigator 21 Software (Thermo Fisher Scientific Inc.). In addition, both temperature and pH were measured every second day, and oxygen concentration three times over the duration of the experiment in all replicates. Water temperature was measured using a calibrated glass thermometer (certified by Physikalisch-Technische Bundesanstalt (PTB), Braunschweig, Germany) and pH_NBS_ using a hand-held pH meter (Orion Star Plus™ 3-Star, Thermo Fisher Scientific Inc., Beverly, USA). Oxygen concentration was measured using an O_2_ meter and probe (Oxi 330i/SET, WTW, Weilheim, Germany). The salinity of the intake water was recorded every 5 min during the entire duration of the experiments using a CT-probe (Aqua TROLL 100^®^) with Win-Situ 5 data acquisition software (In-Situ Inc., Collins, USA). Sea water samples for alkalinity measurements were collected three times during the sea urchin experiments and five times during the shrimp experiment respectively. Total alkalinity (TA) was estimated using high-precision potentiometric titration (Haraldsson, Anderson et al. 1997). CO2SYS (Pierrot et al 2006) was used to calculate the saturation state for aragonite and calcite (Ω_arg_ and Ω_cal_), dissolved inorganic carbon (DIC) and concentrations of carbonate [CO_3_^2-^] and bicarbonate [HCO_3_^-^] using total alkalinity, pH, temperature and salinity with the dissociation constants from Mehrbach et al (1973) refitted by Dickson and Millero (1987). Results are presented in Table S1 as minimum, maximum and mean measured values ± standard deviation (SD). Mean pH_NBS_ level (and SD) in each aquarium was calculated using the actual [H^+^] concentration and back calculated to pH_NBS_.

The oil-water dispersion was generated by a mechanical dispersion of oil using a mixing valve connected to a continuous flow system (CFS, Sanni et al., 1998). Oil was placed at the top of a two-compartment glass cylinder separated by a Teflon piston. At the bottom of the cylinder, distilled water pressurized by a HPLC pump was used to push the piston upwards at the desired speed to deliver oil at the same rate into the continuous flow of seawater. The 5 mg L^-1^ dispersion was pumped into one header tanks (10 L) and then pumped by peristaltic pumps (model 520, Watson and Marlow, Cornwall, UK) into either header tanks or aquaria and diluted with seawater to achieve nominal concentrations of 0.5 mg L^-1^ (see explanatory figure S5).

Determination of polycyclic hydrocarbon (PAH) in seawater was carried out using gas chromatography with mass spectrometric detection (GC-MS). The analyses included the 16 EPA PAHs and the alkylated congeners (C1 to C2/C3) of naphthalene, phenanthrene, chrysene and dibenzothiphene. Water samples for the PAH analysis were collected 7 times during sea urchin experiments and 3 times during the shrimp experiment. Samples from the non-oil exposed aquaria were sampled as collective samples from all the aquaria in the treatment to ensure that none of the aquaria was contaminated. After addition of the appropriate amount of standards (Naftalen-d8, Dibenzothiofen-d8, Fenantren-d10, Fluoranten-d10, Pyren-d10, Krysen-d10, Benzo(a)pyren-d12, Dibenzo(ah)antracen-d14, Acenaftylen-d8) the samples were extracted with cyclohexane. Thereafter the sample was transferred to vials for instrumental analysis by Gas Chromatography (HP5890, Hewlett Packard, USA) connected to a Mass Spectrometer (Finnigan SSQ7000, USA) and analysed in selected ion mode (GC/MS-SIM) as described previously (Baussant et al., 2001; Jonsson et al., 2004). The quantification limit for each PAH measured in seawater was based on EPA method 610 and the Douglas method (Douglas et al., 1994). For seawater, detection limit ranged between 0.005 and 0.01 µg L^-1^. Alkylated homologues were quantitated based on the response factors of the parent compound standards.

**Results**

**Seawater carbonate chemistry and temperature.** Although some significant differences in temperature were detected between aquaria for Experiment 1 (One–way ANOVA, F_23,71_ = 4.22, p < 0.0001), the actual magnitude of this difference was small, i.e. less than 0.1 °C. No significant differences were detected in temperature between aquaria for Experiment 2 (One–way ANOVA, F_23,161_ = 1.20, p = 0.260). Mean temperatures for the shrimp experiment were 6.7 °C in the controls and 9.5 °C in the OAW treatments with no significant difference detected between the replicates (One-way ANOVA, F_1,11_ = 0.77, p = 0.787).

No significant differences in water alkalinity were detected between treatments in the sea urchin experiment (TA = 2306 ± 14 µmol kg^-1^, One-way ANOVA, F_1,9_ = 0.06, p = 0.803). In the pH treatments, pH_NBS_ fluctuated less than 1 % from the mean values. Sea water was under saturated with respect to aragonite at pH_NBS_ < 7.6 (Table S1).

In the shrimp experiment, temperature had no significant difference on the mean pH_NBS_ (One-way ANOVA, F_1,11_ = 0.81, p = 0.559) and mean pH_NBS_ were 8.05 and 7.60 respectively. The mean total alkalinity was 2305 ± 12 µmol kg^-1^ with no differences between the pH treatments (Mann-Whitney U, p = 0.841). Sea water was under saturated with respect to aragonite when pH_NBS_ was below 7.6 (Table S1). Oxygen concentration in the experiments varied between 9.3 and 9.7 mg L^-1^.

Sanni, S., Øysæd, K. B., Høivangli, V. & Gaudebert, B. A Continuous Flow System (CFS) for chronic exposure of aquatic organisms. *Mar. Environ. Res.* **46**, 97-101, doi:http://dx.doi.org/10.1016/S0141-1136(97)00086-X (1998).

**Supplementary figures and tables**

**
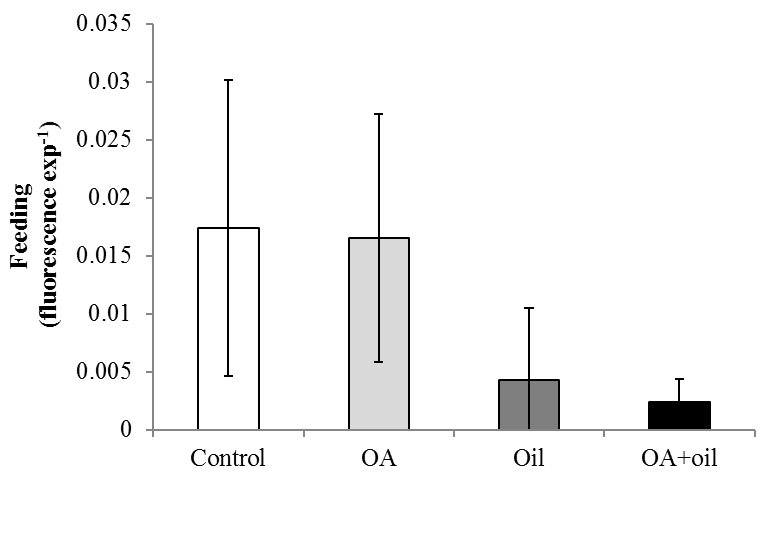
**

**Figure S1.** The effect of global drivers (pH) and Oil on early (day 8-12 post hatch) larval *Strongylocentrotus droebachiensis* on feeding rate. Control (pH 8.0, 6.7 ºC, white), Oil (pH 8.0, 6.7 ºC + Oil, dark grey), OA (pH 7.6, 9.5 ºC, light grey), OA + Oil (pH 7.6, 9.5 ºC + Oil, black). Six replicates for each treatment. Data are presented as mean values ± SD.

**
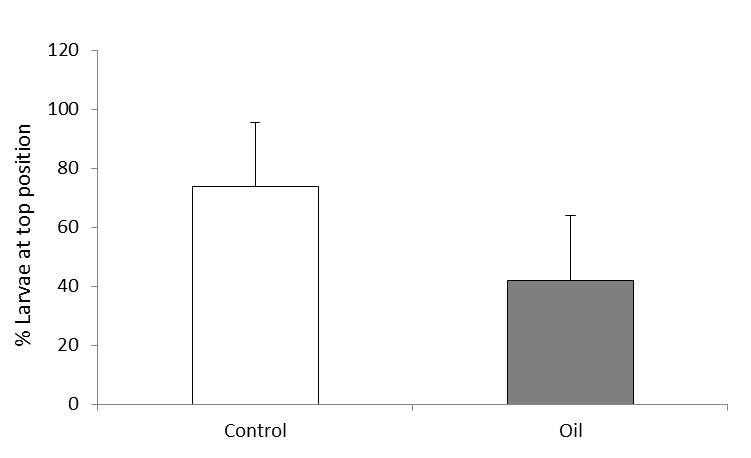
**

**Figure S2.** The effect of oil on larval *Pandalus borealis* swimming. (pH 8.0, 6.7 ºC, white), Oil (pH 8.0, 6.7 ºC + Oil, light grey) Six replicates for each treatment. Data are presented as mean values ± SD.

**
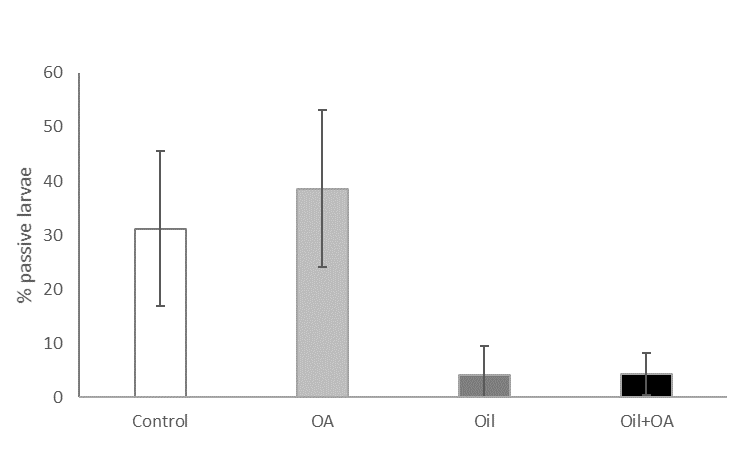
**

**Figure S3.** The effect of oil on larval *Strongylocentrotus droebachiensis* activity during oil exposure day 23. Control (pH 8.0, 9.5 ºC, white), OA (pH 7.6, 9.5 ºC light grey), Oil (pH 8.0, 9.5 ºC + Oil dark grey), Oil+OA (pH 7.6, 9.5 ºC + Oil dark grey), Six replicates for each treatment. Data are presented as mean values ± SD.

**
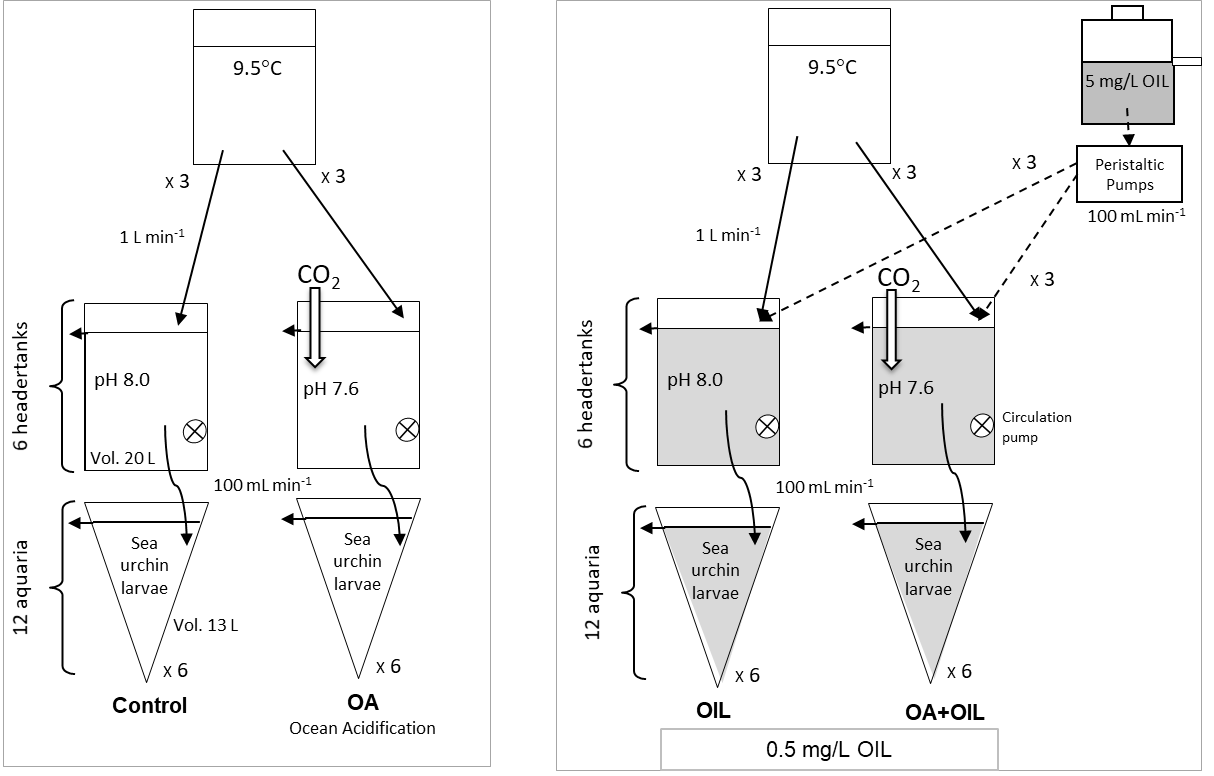
**

**Figure S4.** Experimental set up for the sea urchin experiments. There was a total of four treatments, Control (pH_NBS_ 8.0, no oil), OA (pH_NBS_ 7.6, no oil), Oil (pH_NBS_ 8.0, oil) and OA+Oil (pH_NBS_ 7.6, oil)

**
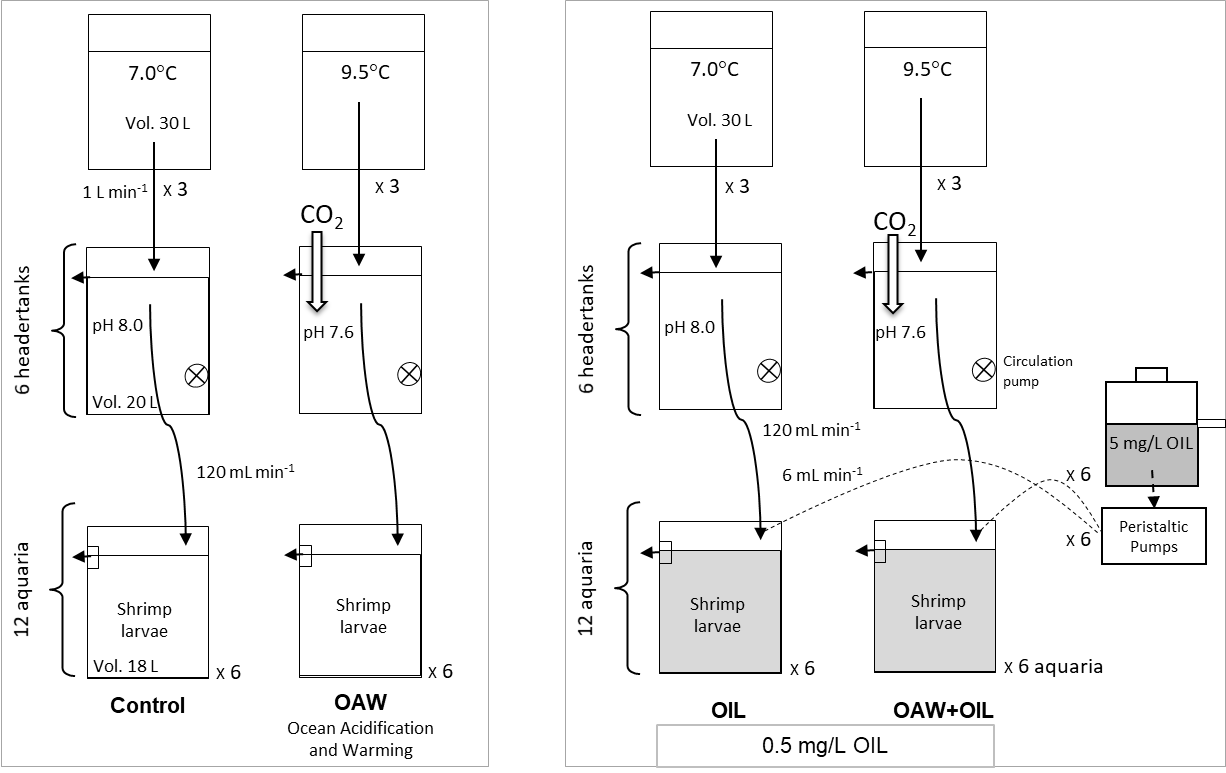
**

**Figure S5.** Experimental set up for the shrimp experiment. There was a total of four treatments, Control (pH_NBS_ 8.0, 6.7 °C, no oil), OAW (pH_NBS_ 7.6, 9.5 °C, no oil), Oil (pH_NBS_ 8.0, 6.7 °C, no oil) and OAW+Oil (pH_NBS_ 7.6, 9.5 °C, no oil).

**
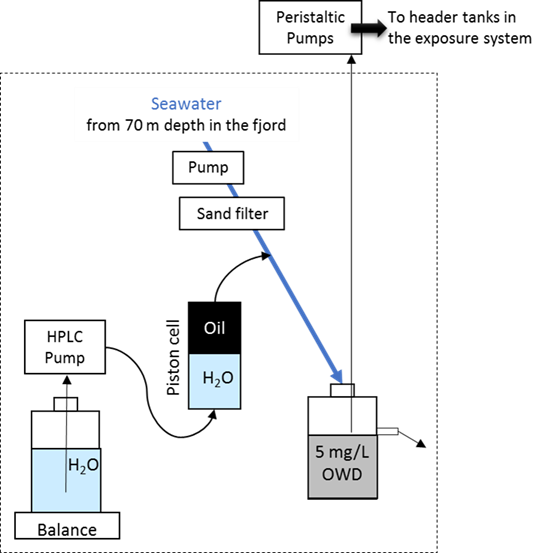
**

**Figure S6.** Explanatory figure of the production of mechanically dispersed oil. The oil-water dispersion (OWD) was generated by a mechanical dispersion of oil using a mixing valve connected to a continuous flow system.

**
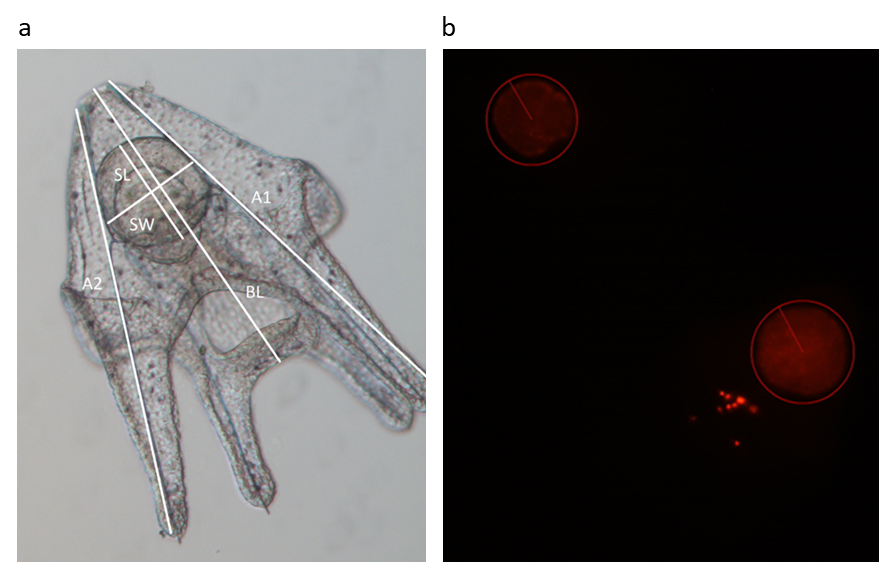
**

**Figure S7 a-b.** Three morphometric parameters (body length (BL) and posterolateral rod lengths (POL) (A1=POL1, A2=POL2)) were measured for each larva using Image J software. In addition to the area of larval the stomach (stomach length (SL), stomach width (SW)) and stomach fluorescence were measured.

**Table S1** Physiochemical conditions of seawaters during the three experiments, (exp. 1. Sea urchin 18 d oil and OA exposure, exp. 2. Sea urchin 44 d oil and OA exposure, and exp. 3. Shrimp 30 d oil and OA/OW exposure). pH_NBS_; pH NBS scale, A_T_; total alkalinity, DIC; total dissolved inorganic carbon, *p*CO_2_; partial pressure of CO_2_. For temperature mean values (± SD) are provided, for all the calculated variables range of values are provided.

| **Incubation group** | **Temperature (°C)** | **pH_NBS_** | **CO_2_ (uatm)** | **DIC** | **A_T_** |
| --- | --- | --- | --- | --- | --- |
| *Experiment 1*  *(Sea urchin)* | | | | | |
| Control | 9.41  (± 0.03) | 8.03  (7.98 - 8.08) | 555  (489 - 634) | 2183  (2166-2199) | 2306  (± 14) |
| OA | 9.41  (± 0.03) | 7.64  (7.59 - 7.68) | 1455  (1323 – 1646) | 2307  (2283-2320) | 2306  (± 14) |
| Oil | 9.40  (± 0.11) | 8.06  (8.03 - 8.09) | 515  (476 – 559) | 2172  (2170-2176) | 2306  (± 14) |
| OA + Oil | 9.52  (± 0.08) | 7.64  (7.61 - 7.68) | 1455  (1323 -1569) | 2306  (2305-2307) | 2306  (± 14) |
| *Experiment 2*  *(Sea urchin)* | | | | | |
| Control | 9.41  (± 0.03) | 8.02  (7.98 - 8.07) | 569  (502 - 634) | 2188  (2178-2193) | 2306  (± 14) |
| OA | 9.41  (± 0.03) | 7.63  (7.59 – 7.68) | 1485  (1318 – 1640) | 2307  (2283-2320) | 2306  (± 14) |
| Oil | 9.40  (± 0.11) | 8.03  (7.99 - 8.07) | 555  (501 – 618) | 2184  (2178-2190) | 2306  (± 14) |
| OA + Oil | 9.52  (± 0.08) | 7.64  (7.59 – 7.69) | 1455  (1292 – 1646) | 2307  (2291-2333) | 2306  (± 14) |
| *Experiment 3*  *(Shrimp)* | | | | | |
| Control | 6.7  (± 0.08) | 8.05  (8.03-8.08) | 511  (470-537) | 2185  (2180-2189) | 2305  (± 12) |
| OA/OW | 9.5  (± 0.07) | 7.59  (7.55-7.67) | 1627  (1354-1795) | 2206  (2199-2209) | 2305  (± 12) |
| Oil | 6.7  (± 0.07) | 8.05  (8.03-8.08) | 511  (470-537) | 2187  (2180-2189) | 2305  (± 12) |
| OA/OW + Oil | 9.5  (± 0.06) | 7.60  (7.56-7.67) | 1589  (1349-1753) | 2315  (2306-2318) | 2305  (± 12) |

**Table S2.** Mean concentration (± SD) of PAHs (µg L^-1^) in seawater samples from the control and OA/OW Oil exposed aquaria. The samples were taken during the experiment (n = 2) for each treatment. Liquid/liquid extraction method was used for analysing the PAHs. nd = not detected. SC = shrimp control, UC = sea urchin control, SOIL = shrimp Oil, SOA/OW + Oil = shrimp OA/OW + Oil, U1Oil = Sea urchin exp.1 Oil, U1OA + Oil = Sea urchin exp.1 OA + Oil, U2Oil = Sea urchin exp. 2 Oil and U2OA + Oil = Sea urchin exp.2 OA + Oil.

| **Chemical (µg L-1)** | **SC** | **UC** | **S Oil** | **SOA/OW+Oil** | | **U1 Oil** | **U1 OA+Oil** | **U2 Oil** | **U2 OA+Oil** |
| --- | --- | --- | --- | --- | --- | --- | --- | --- | --- |
| Naphthalene | nd | nd | 0.299 ± 0.008 | | 0.335 ± 0.025 | 0.435 ± 0.0295 | 0.450 ± 0.0566 | 0.445 ± 0.1595 | 0.338 ± 0.0347 |
| C1-Naphthalene | nd | 0.0018 | 1.028 ± 0.015 | | 1.143 ± 0.092 | 0.493 ± 0.0361 | 0.539 ± 0.0682 | 0.523 ± 0.1869 | 0.3867 ± 0.0396 |
| C2-Naphthalene | nd | nd | 1.785 ± 0.066 | | 2.014 ± 0.143 | 1.698 ± 0.1466 | 1.916 ± 0.2808 | 1.797 ± 0.6419 | 1.3212 ± 0.1224 |
| C3-Naphthalene | nd | nd | 1.391 ± 0.041 | | 1.597 ± 0.088 | 1.4279 ± 0.1517 | 1.692 ± 0.4612 | 1.468 ± 0.5019 | 1.0642 ± 0.0969 |
| Sum 2 ring PAH | - | 0.0018 | 4.503 | | 5.089 | 4.054 | 4.616 | 4.233 | 3.111 |
| Fluorene | nd | nd | 0.025 ± 0.001 | | 0.026 ± 0.001 | 0.039 ± 0.0040 | 0.040 ± 0.0068 | 0.046 ±0.0167 | 0.032 ± 0.003 |
| Phenanthrene | nd | nd | 0.060 ± 0.004 | | 0.068 ± 0.007 | 0.090 ± 0.0086 | 0.095 ± 0.0070 | 0.103 ± 0.0388 | 0.074 ±0.004 |
| C1-Phen/Anthr | nd | nd | 0.107 ± 0.004 | | 0.122 ± 0.015 | 0.139 ± 0.0143 | 0.167 ± 0.0429 | 0.166 ± 0.0580 | 0.118 ± 0.0068 |
| C2-Phen/Anthr | nd | nd | 0.123 ± 0.005 | | 0.146 ± 0.009 | 0.164 ± 0.0201 | 0.234 ± 0.136 | 0.203 ± 0.006 | 0.150 ± 0.0138 |
| Sum 3 ring PAH | - | - | 0.315 | | 0.362 | 0.463 | 0.582 | 0.561 | 0.406 |
| Dibenzothiophene | nd | 0.0007 | 0.010 ± 0.001 | | 0.011 ± 0.001 | 0.016 ± 0.0014 | 0.017 ± 0.0023 | 0.018 ± 0.0077 | 0.014 ± 0.0006 |
| C1-Dibenzothiophene | nd | nd | 0.028 ± 0.002 | | 0.035 ± 0.0053 | 0.038 ± 0.007 | 0.058 ± 0.0286 | 0.047 ± 0.0164 | 0.035 ± 0.0053 |
| C2-Dibenzothiophene | nd | nd | 0.029 ± 0.001 | | 0.036 ± 0.004 | 0.014 ± 0.0016 | 0.023 ± 0.0198 | 0.216 ± 0.0083 | 0.016 ± 0.0029 |
| Sum DBTs | - | 0.0007 | 0.067 | | 0.08 | 0.126 | 0.192 | 0.179 | 0.124 |
| **Total PAHs** | **-** | **0.0025** | **4.895 ± 0.147** | | **5.545 ± 0.320** | **4.628 ± 0.353** | **5.340 ± 1.157** | **4.944 ±1.760** | **3.620 ± 0.337** |

**Table S3.** Results of multiple ANOVA testing for the effect of OA/OW and Oil, and their interaction on different parameters of the *Pandalus borealis* and *Strongylocentrotus droebachiensis* larvae. Degrees of freedom (df), mean of square (MS), F-ratio (F), probability level (*p*).

| **Parameter** | **Source** | | **df** | **F** | **p** |
| --- | --- | --- | --- | --- | --- |
| *Shrimp larvae* | | | | | |
| Mortality | | OA/OW  Oil  OA/OW*Oil | 3  3  3 | 8.54  0.25  0.16 | 0.008  0.2  0.69 |
| Feeding rate | | OA/OW  Oil  OA/OW*Oil | 3  3  3 | 3.26  9.63  0.59 | 0.08  0.004  0.45 |
| Growth/size | | OA/OW  Oil  OA/OW*Oil | 3  3  3 | 137.91  62.25  0.54 | <0.0001  <0.0001  0.46 |
| Swimming index | | OA/OW  Oil  OA/OW*Oil | 3  3  3 | 83.94  0.31  0.44 | <0.0001  0.58  0.52 |
| The abnormality index | | OA/OW  Oil  OA/OW*Oil | 3  3  3 | 12.10  0.92  1.69 | 0.92  0.002  0.21 |
| Respiration | |  | 3,20 | 2.43 | 0.10 |
| *Sea Urchin larvae* | | | | | |
| Experiment 1 | | | | | |
| Mortality | | OA  Oil  OA*Oil | 3  3  3 | 0.04  65.45  0.01 | 0.84  <0.0001  0.93 |
| Feeding rate | | OA  Oil  OA*Oil | 3  3  3 | 1.00  95.87  0.15 | 0.32  <0.0001  0.70 |
| Growth/size | | OA  Oil  OA*Oil | 3  3  3 | 21.92  21.92  0.77 | <0.0001  <0.0001  0.77 |
| Symmetry Index | |  | 23,560 | 1.53 | 0.054 |
| Experiment 2 | | | | | |
| Mortality | | Model | 3,18 | 0.02 | p>0.99 |
| Feeding rate | | OA  Oil  OA*Oil | 2  2  2 | 18.08  91.23  5.57 | <0.0001  <0.0001  0.02 |
| Growth/size | | Model  OA  Oil  OA*Oil | 3,23  3  3  3 | 8.51  6.97  16.73  1.85 | 0.0008  0.0157  0.0006  0.19 |
| Swimming index | | OA  Oil  OA*Oil | 3  3  3 | 4.50  16.52  3.34 | 0.041  0.0002  0.09 |
| Symmetry index | |  | 23,3524 | 0.64 | 0.054 |

**Table S4** Respiration rate growth rates (RR GR in pmol O_2_ ind^-1^ h^-1^ μm^-1^_BL_) for sea urchins in exp. 2, were calculated as the coefficient of the significant linear relationship between respiration rate (pmol O_2_ ind^-1^ h^-1^) and body length (BL) (μm). Results of the regressions (Intercept, p-value, R^2^, F-value and df: degree of freedom) are given for each treatment.

| Treatment | RR GR | df | F-value | Intercept | R^2^ | p-value |
| --- | --- | --- | --- | --- | --- | --- |
| Control | 2.56 | 16 | 14.20 | -653.77 | 48.63 | 0.0019 |
| OA | 2.60 | 20 | 33.22 | -651.74 | 63.61 | <0.0001 |
| Oil | 1.82 | 20 | 11.16 | -470.82 | 37.00 | 0.0034 |
| OA + Oil | 2.94 | 16 | 21.05 | -762.18 | 58.39 | 0.0004 |

**Table S5** Larval mortality rate (MR in % day^-1^) in sea urchin exp.1 were calculated as the coefficient of the significant linear relationship between mortality and time post fertilization (tpf). Results of the regressions (Intercept, p-value, R^2^, F-value and df: degree of freedom) are given for each culture replicate with corresponding treatment. Data in bold (p > 0.05) were removed from subsequent analyses.

| **Treatment** | **Replicate** | **MR** | **df** | **F-value** | **Intercept** | **R^2^** | **p-value** |
| --- | --- | --- | --- | --- | --- | --- | --- |
| Control | 1 | 1.53 | 15 | 16.29 | 0.25 | 0.54 | 0.0012 |
|  | **2** | **0.95** | **15** | **1.60** | **0.05** | **0.10** | **0.23** |
|  | 3 | 2.50 | 15 | 41.09 | -0.035 | 0.75 | <0.0001 |
|  | 4 | 2.41 | 15 | 9.67 | 0.18 | 0.41 | 0.008 |
|  | **5** | **0.98** | **15** | **2.19** | **0.074** | **0.14** | **0.16** |
|  | 6 | 2.63 | 15 | 43.12 | -0.035 | 0.75 | <0.0001 |
| OA/OW | 1 | 2.24 | 15 | 11.80 | 0.079 | 0.46 | 0.004 |
|  | 2 | 2.96 | 15 | 28.86 | -0.0072 | 0.67 | <0.0001 |
|  | 3 | 1.90 | 15 | 15.94 | -0.0080 | 0.53 | 0.0013 |
|  | 4 | 2.11 | 15 | 13.38 | 0.013 | 0.49 | 0.0026 |
|  | 5 | 1.32 | 15 | 6.77 | 0.11 | 0.33 | 0.021 |
|  | **6** | **1.23** | **14** | **2.00** | **0.14** | **0.13** | **0.19** |
| Oil | 1 | 7.08 | 15 | 304.73 | -0.12 | 0.96 | <0.0001 |
|  | 2 | 3.92 | 15 | 27.88 | -0.033 | 0.67 | <0.0001 |
|  | 3 | 7.22 | 15 | 157.46 | -0.16 | 0.92 | <0.0001 |
|  | 4 | 5.00 | 15 | 73.67 | -0.079 | 0.84 | <0.0001 |
|  | 5 | 4.43 | 15 | 41.54 | -0.075 | 0.75 | <0.0001 |
|  | 6 | 4.84 | 15 | 9.88 | 0.071 | 0.41 | 0.0072 |
| OA/OW + Oil | 1 | 4.32 | 15 | 68.34 | -0.018 | 0.83 | <0.0001 |
|  | 2 | 6.40 | 15 | 90.79 | -0.10 | 0.87 | <0.0001 |
|  | 3 | 6.43 | 15 | 96.99 | 0.037 | 0.87 | <0.0001 |
|  | 4 | 7.02 | 15 | 96.01 | -0.11 | 0.87 | <0.0001 |
|  | 5 | 4.21 | 15 | 25.88 | -0.12 | 0.65 | 0.0002 |
|  | 6 | 4.41 | 14 | 25.26 | 0.014 | 0.66 | 0.0002 |

**Table S6** Larval mortality rate (MR in % day^-1^) in sea urchin exp. 2 were calculated as the coefficient of the significant linear relationship between mortality and time post fertilization (tpf). Results of the regressions (Intercept, p-value, R^2^, F-value and df: degree of freedom) are given for each culture replicate with corresponding treatment. Data in bold (p > 0.05) were removed from subsequent analyses.

| **Treatment** | **Replicate** | **MR** | **df** | **F-value** | **Intercept** | **R^2^** | **p-value** |
| --- | --- | --- | --- | --- | --- | --- | --- |
| Control | 1 | 1.1 | 24 | 19.66 | 0.05 | 0.46 | 0.0002 |
|  | **2** | **0.16** | **24** | **0.27** | **0.10** | **0.012** | **0.61** |
|  | 3 | 2.6 | 24 | 108.22 | -0.053 | 0.82 | <0.0001 |
|  | 4 | 2.3 | 24 | 40.33 | 0.18 | 0.64 | <0.0001 |
|  | 5 | 2.3 | 24 | 38.72 | -0.035 | 0.63 | <0.0001 |
|  | 6 | 2.1 | 24 | 114.39 | 0.0026 | 0.83 | <0.0001 |
| OA/OW | 1 | 2.6 | 24 | 79.44 | 0.051 | 0.78 | <0.0001 |
|  | 2 | 2.9 | 24 | 108.80 | -0.002 | 0.83 | <0.0001 |
|  | 3 | 2.4 | 24 | 70.77 | -0.058 | 0.75 | <0.0001 |
|  | 4 | 1.9 | 24 | 30.55 | 0.033 | 0.57 | <0.0001 |
|  | 5 | 0.79 | 24 | 11.54 | 0.15 | 0.33 | 0.0025 |
|  | 6 | 2.2 | 24 | 58.79 | 0.076 | 0.74 | <0.0001 |
| Oil | 1 | 1.4 | 24 | 55.00 | 0.030 | 0.71 | <0.0001 |
|  | **2** | **0.069** | **24** | **0.05** | **0.11** | **0.0020** | **0.83** |
|  | 3 | 2.4 | 24 | 93.17 | -0.040 | 0.80 | <0.0001 |
|  | 4 | 1.8 | 24 | 23.78 | 0.22 | 0.51 | <0.0001 |
|  | 5 | 2.8 | 24 | 44.40 | -0.077 | 0.66 | <0.0001 |
|  | 6 | 1.7 | 24 | 48.85 | 0.030 | 0.68 | <0.0001 |
| OA/OW + Oil | 1 | 2.8 | 24 | 68.82 | 0.028 | 0.75 | <0.0001 |
|  | 2 | 2.6 | 24 | 125.07 | 0.020 | 0.84 | <0.0001 |
|  | 3 | 3.2 | 24 | 76.16 | -0.12 | 0.77 | <0.0001 |
|  | 4 | 1.5 | 24 | 38.70 | 0.062 | 0.63 | <0.0001 |
|  | 5 | 0.89 | 24 | 15.38 | 0.14 | 0.40 | 0.0007 |
|  | 6 | 1.6 | 24 | 27.33 | 0.11 | 0.54 | <0.0001 |

**Table S7** Results of regression analysis of shrimp mortality in *P. borealis* larvae raised in control (pH 8.1), OA/OW (7.6), Oil (pH 8.1 + Oil conc. 0.5 mg L^-1^) and OA/OW + Oil (pH 7.6 + Oil conc. 0.5 mg L^-1^).

| **Treatment** | **Replicate** | **MR** | **df** | **F-value** | **Intercept** | **R^2^** | **p-value** |
| --- | --- | --- | --- | --- | --- | --- | --- |
| Control | 1 | 2.61 | 24 | 132.48 | 113.03 | 0.85 | <0.0001 |
|  | 2 | 1.27 | 24 | 382.75 | 103.78 | 0.95 | <0.0001 |
|  | 3 | 0.45 | 24 | 513.13 | 101.12 | 0.96 | <0.0001 |
|  | 4 | 0.67 | 24 | 277.48 | 101.66 | 0.94 | <0.0001 |
|  | 5 | 0.60 | 24 | 403.92 | 101.51 | 0.95 | <0.0001 |
|  | 6 | 1.17 | 24 | 571.51 | 100.85 | 0.96 | <0.0001 |
| OA/OW | 1 | 1.44 | 24 | 289.89 | 103.77 | 0.94 | <0.0001 |
|  | 2 | 0.98 | 24 | 312.23 | 102.47 | 0.95 | <0.0001 |
|  | 3 | 1.60 | 24 | 412.48 | 101.24 | 0.96 | <0.0001 |
|  | 4 | 2.53 | 24 | 100.44 | 99.93 | 0.85 | <0.0001 |
|  | 5 | 2.34 | 24 | 175.52 | 101.74 | 0.90 | <0.0001 |
|  | 6 | 1.35 | 24 | 276.27 | 101.26 | 0.94 | <0.0001 |
| Oil | 1 | 1.53 | 24 | 637.42 | 104.21 | 0.97 | <0.0001 |
|  | 2 | 0.30 | 24 | 516.64 | 99.72 | 0.96 | <0.0001 |
|  | 3 | 0.98 | 24 | 443.23 | 97.78 | 0.95 | <0.0001 |
|  | 4 | 0.61 | 24 | 241.36 | 101.71 | 0.93 | <0.0001 |
|  | 5 | 1.18 | 24 | 523.96 | 102.49 | 0.96 | <0.0001 |
|  | 6 | 0.68 | 24 | 290.24 | 100.65 | 0.94 | <0.0001 |
| OA/OW + Oil | 1 | 3.54 | 24 | 272.93 | 102.53 | 0.94 | <0.0001 |
|  | 2 | 1.54 | 24 | 395.46 | 104.80 | 0.95 | <0.0001 |
|  | 3 | 1.85 | 24 | 134.39 | 98.06 | 0.88 | <0.0001 |
|  | 4 | 1.30 | 24 | 263.29 | 99.97 | 0.93 | <0.0001 |
|  | 5 | 1.51 | 24 | 894.72 | 98.20 | 0.98 | <0.0001 |
|  | 6 | 0.80 | 24 | 172.38 | 99.40 | 0.88 | <0.0001 |

**Table S8** Body length growth rates (BL GR in μm ln(day)^-1^) of sea urchin exp. 1 were calculated as the coefficient of the significant logarithmic relationship between BL and time post fertilization (tpf). Results of the regressions (Intercept, p-value, R^2^, F-value and df: degree of freedom) are given for each culture replicate with corresponding treatment.

| **Treatment** | **Replicate** | **BL GR** | **df** | **F-value** | **Intercept** | **R^2^** | **p-value** |
| --- | --- | --- | --- | --- | --- | --- | --- |
| Control | 1 | 77.99 | 120 | 463.21 | 185.55 | 79.56 | <0.0001 |
|  | 2 | 74.65 | 126 | 558.53 | 186.59 | 81.71 | <0.0001 |
|  | 3 | 74.78 | 129 | 297.40 | 188.47 | 69.91 | <0.0001 |
|  | 4 | 73.59 | 101 | 381.27 | 185.84 | 79.22 | <0.0001 |
|  | 5 | 76.88 | 135 | 362.85 | 190.52 | 73.03 | <0.0001 |
|  | 6 | 78.90 | 124 | 393.56 | 183.32 | 76.19 | <0.0001 |
| OA/OW | 1 | 72.22 | 122 | 151.70 | 187.50 | 55.63 | <0.0001 |
|  | 2 | 78.67 | 129 | 377.54 | 173.23 | 74.68 | <0.0001 |
|  | 3 | 76.11 | 101 | 360.12 | 177.15 | 78.27 | <0.0001 |
|  | 4 | 65.73 | 118 | 382.24 | 193.24 | 76.56 | <0.0001 |
|  | 5 | 67.39 | 126 | 685.55 | 181.48 | 84.58 | <0.0001 |
|  | 6 | 77.52 | 90 | 363.89 | 171.24 | 80.35 | <0.0001 |
| Oil | 1 | 66.49 | 45 | 200.78 | 188.23 | 82.02 | <0.0001 |
|  | 2 | 66.86 | 56 | 95.14 | 197.32 | 63.37 | <0.0001 |
|  | 3 | 71.93 | 92 | 625.57 | 178.05 | 87.30 | <0.0001 |
|  | 4 | 67.95 | 78 | 346.42 | 181.72 | 0.82 | <0.0001 |
|  | 5 | 65.89 | 128 | 320.72 | 205.69 | 0.72 | <0.0001 |
|  | 6 | 77.07 | 126 | 524.53 | 167.58 | 0.81 | <0.0001 |
| OA/OW + Oil | 1 | 64.49 | 82 | 161.28 | 169.18 | 66.57 | <0.0001 |
|  | 2 | 63.26 | 64 | 207.02 | 178.59 | 76.67 | <0.0001 |
|  | 3 | 60.79 | 100 | 183.87 | 189.84 | 65.00 | <0.0001 |
|  | 4 | 70.29 | 28 | 202.78 | 169.85 | 88.25 | <0.0001 |
|  | 5 | 55.60 | 71 | 131.88 | 198.96 | 65.33 | <0.0001 |
|  | 6 | 63.87 | 107 | 255.96 | 187.16 | 70.71 | <0.0001 |

**Table S9** Body length growth rates (BL GR in μm ln (day)^-1^) of sea urchin exp. 2 were calculated as the coefficient of the significant logarithmic relationship between BL and time post fertilization (tpf). Results of the regressions (Intercept, p-value, R^2^, F-value and df: degree of freedom) are given for each culture replicate with corresponding treatment.

| **Treatment** | **Replicate** | **BL GR** | **df** | **F-value** | **Intercept** | **R^2^** | **p-value** |
| --- | --- | --- | --- | --- | --- | --- | --- |
| Control | 1 | 77.28 | 258 | 904.98 | 182.87 | 77.88 | <0.0001 |
|  | 2 | 79.51 | 251 | 1254.82 | 176.84 | 83.39 | <0.0001 |
|  | 3 | 90.80 | 248 | 879.31 | 155.58 | 78.07 | <0.0001 |
|  | 4 | 79.19 | 203 | 713.60 | 190.14 | 77.93 | <0.0001 |
|  | 5 | 79.92 | 269 | 823.41 | 183.61 | 75.44 | <0.0001 |
|  | 6 | 82.52 | 253 | 943.58 | 176.36 | 78.92 | <0.0001 |
| OA/OW | 1 | 79.47 | 256 | 625.30 | 155.43 | 71.03 | <0.0001 |
|  | 2 | 80.15 | 278 | 708.96 | 146.95 | 71.91 | <0.0001 |
|  | 3 | 69.57 | 236 | 644.11 | 181.53 | 73.27 | <0.0001 |
|  | 4 | 64.17 | 250 | 887.16 | 194.27 | 78.08 | <0.0001 |
|  | 5 | 67.36 | 264 | 945.93 | 180.49 | 78.24 | <0.0001 |
|  | 6 | 67.21 | 222 | 646.16 | 184.40 | 74.51 | <0.0001 |
| Oil | 1 | 73.18 | 270 | 723.27 | 189.78 | 73.89 | <0.0001 |
|  | 2 | 59.62 | 254 | 601.46 | 207.01 | 70.39 | <0.0001 |
|  | 3 | 68.99 | 264 | 556.13 | 196.34 | 67.89 | <0.0001 |
|  | 4 | 62.75 | 257 | 707.45 | 199.48 | 73.43 | <0.0001 |
|  | 5 | 75.96 | 287 | 470.07 | 187.64 | 62.17 | <0.0001 |
|  | 6 | 65.20 | 263 | 538.13 | 203.00 | 67.26 | <0.0001 |
| OA/OW + Oil | 1 | 68.53 | 268 | 387.17 | 190.22 | 59.19 | <0.0001 |
|  | 2 | 68.40 | 245 | 527.86 | 185.56 | 68.39 | <0.0001 |
|  | 3 | 65.59 | 244 | 602.68 | 187.09 | 71.27 | <0.0001 |
|  | 4 | 71.90 | 258 | 646.76 | 179.92 | 71.56 | <0.0001 |
|  | 5 | 56.54 | 265 | 799.93 | 198.30 | 75.19 | <0.0001 |
|  | 6 | 55.10 | 219 | 286.54 | 206.30 | 56.79 | <0.0001 |
